# Sex, age, and seasonal distribution characteristics of bacterial and fungal infections

**DOI:** 10.1371/journal.pone.0355413

**Published:** 2026-08-05

**Authors:** Qinxingzi Li, Zhongzhi Liang, Zeling Dong, Jialin Xiang, Qiongyao Li, Tiantian Wang, Daimin Xiao, Zuyi Chen

**Affiliations:** 1 Department of Laboratory Medicine, The second affiliated hospital of Zunyi medical university, Zunyi, Guizhou, P.R. China; 2 Department of Laboratory Medicine, Affiliated Hospital of Zunyi Medical University, Zunyi, Guizhou, P.R. China; 3 Information Division, Affiliated Hospital of Zunyi Medical University, Zunyi, Guizhou, P.R. China; Children’s National Hospital, George Washington University, UNITED STATES OF AMERICA

## Abstract

**Objective:**

Studies on the common distribution characteristics of multiple bacterial and fungal infections across sex, age groups, and seasons remain scarce. This study aimed to analyze the epidemiological distribution patterns of common bacteria and fungi in the above three dimensions.

**Methods:**

Retrospectively collected bacterial and fungal testing data from a grade A tertiary general hospital between March 2016 and February 2022. A total of 51 microbial species with the highest detection frequency were enrolled, accounting for 97.35% of all positive samples. Their distribution differences in sex, age group, season, and Gram staining classification were analyzed.

**Results:**

Among the 51 common bacteria and fungi, 40 exhibited significant sex-related differences, with 38 showing the highest proportion in males and only 2 in females (P < 0.001). 48 species had significant age-related differences, with no significant disparity in the number of pathogens peaked across different age groups. Seasonal distribution differences were significant in 32 species, of which 20 reached the peak prevalence in summer (P < 0.001). The proportion of Gram-positive bacteria affected by sex (15/22) and season (10/22) was significantly lower than that of Gram-negative bacteria (sex: 22/24, P = 0.045; season: 18/24, P = 0.022).

**Conclusion:**

This study summarized the preference characteristics of the 51 most prevalent bacteria and fungi infection in terms of sex, age, and season. Compared with Gram-positive bacteria, Gram-negative infections were more susceptible to sex and seasonal temperature variations. Females showed stronger resistance to bacterial and fungal infections, suggesting inherent sex differences in immune function. Bacterial and fungal infections peaked predominantly in summer, highlighting the substantial influence of environmental factors on pathogen infection. These findings indicate that sex, age, and seasonal distribution features should be incorporated into the formulation of infection prevention, diagnosis, and treatment strategies.

## 1 Introduction

Infections caused by bacteria and fungi remain a major threat to human health [1]. For instance, community-acquired pneumonia, a leading cause of infection-related mortality, is predominantly driven by bacterial pathogens [2, 3]. Based on their dependence on the host immune status, pathogenic microorganisms are categorized into two groups: primary pathogens that can directly invade healthy individuals, and opportunistic pathogens that typically trigger infections when host immune function is compromised [4]. In addition, bacteria can be further classified into Gram-positive bacteria and Gram-negative bacteria according to Gram staining characteristics [5]. These two classification frameworks—Gram staining and the primary/opportunistic pathogen distinction—constitute the primary basis for bacterial infection identification [6]; on this groundwork, novel technologies (e.g., PCR, sensor arrays, and whole-genome sequencing) have been developed to further refine detection capabilities [7,8]; Exploring infection distribution patterns under these classifications is critical for formulating effective prevention and control strategies, and also provides an important theoretical basis for investigating infection mechanisms and guiding clinical diagnosis and treatment.

Previous studies have preliminarily summarized the epidemiological characteristics of certain bacterial infections. In terms of sex distribution, *Acinetobacter baumannii* infections are more prevalent in males [9], whereas *Escherichia coli* infections occur more frequently in females [10]. Regarding seasonal variation, the detection rate of *Staphylococcus aureus* fluctuates across seasons: its environmental isolation rate peaks in summer, while higher detection is observed in food samples during winter [11, 12]. In contrast, the incidence of *Escherichia coli* infections rises in warm seasons [13]. The seasonal patterns of some bacterial infections are correlated with the activity cycles of vector insects, which provides insights for exploring transmission mechanisms [14]. With respect to age distribution, the incidence of *Mycobacterium tuberculosis* infections varies among age groups, laying a foundation for research on bacterial evolution and gene mutation [15]. *Haemophilus influenzae* predominantly infects children, and *Klebsiella pneumoniae* mainly affects middle-aged and elderly populations [16]. These characteristics are of great significance for clinical differential diagnosis.

However, most existing studies focus merely on a single pathogen or a single dimension, and comprehensive and systematic analyses integrating the age, sex and seasonal distribution patterns of multiple bacterial and fungal infections remain scarce. Particularly against the backdrop of rising reports on clinical bacterial resistance and associated diseases, research gaps in this field have become increasingly prominent. Accordingly, based on large-sample data, the present study incorporated sex, age and seasonal variables to systematically analyze the epidemiological distribution characteristics of common bacterial and fungal infections. It is expected to provide data support and theoretical references for the formulation of infection prevention and control strategies, optimization of clinical diagnosis and treatment, and exploration of underlying mechanisms.

## 2 Methods

This retrospective study was approved by the Medical Ethics Committee of the Affiliated Hospital of Zunyi Medical University (Ethics Approval Number: KLL-2020–008). The data used in this study were accessed and extracted on 04/06/2023. Data were extracted based on unique medical record numbers associated with patient identification information. All personal identifiable information was anonymized prior to analysis, so informed consent was waived. For each unique medical record number, only laboratory test data from the initial diagnosis were included. All procedures of this study were performed in compliance with the guidelines and regulations formulated by the ethics committee.

The present study was carried out at the Affiliated Hospital of Zunyi Medical University, a major provincial medical center in Guizhou Province. The Department of Clinical Laboratory, where the data were sourced, has obtained accreditation to the International Organization for Standardization 15189 standard. Consecutive sampling was adopted to collect all bacterial and fungal testing results between January 1, 2016 to December 31, 2022. All tests and result reporting were conducted in strict accordance with Chinese health industry standards (WS 288–2008, GB 4789.4–2010, WS/T 489–2016, WS/T 497–2017, WS/T 498–2017, WS/T 499–2017, WS/T 503–2017, WS 288–2017 and WS/T 805–2022). Positive and negative quality control was implemented throughout all testing procedures.

### 2.1 Specimen types

The collection sites of specimens were determined by clinicians based on patients’ infectious manifestations. Only the first eligible specimen obtained at each patient’s visit was included in this study. Specimens from non-open sites encompassed whole blood, pleural effusion, cerebrospinal fluid, puncture fluid, drainage fluid, bile, tissue specimens, bone marrow, dialysate and amniotic fluid. Specimens from open sites contained sputum, secretions, urine, faeces, bronchoalveolar lavage fluid, cervical secretions, catheter specimens, throat swabs, prostatic fluid, semen, gastric juice, gastric contents, breast milk and curettage residues.

### 2.2 Laboratory procedures

All specimens were promptly delivered to the clinical laboratory for primary culture after collection. Culture media were selected according to the types of microorganisms. Aerobic bacteria were inoculated on blood agar plates, chocolate agar plates and MacConkey agar plates. Anaerobic bacteria were cultured on anaerobic blood agar plates and kanamycin-containing anaerobic agar plates. Fungi were grown on Sabouraud Dextrose Agar and CHROMagar chromogenic medium, and brain heart infusion agar was additionally used for deep fungi. *Mycobacterium tuberculosis* was cultured on modified Lowenstein-Jensen medium and MGIT 960 liquid medium. All microbial isolates were finally identified by biochemical assays and mass spectrometry (VITEK MS, bioMérieux, France). Nucleic acid amplification testing (Xpert MTB/RIF, Cepheid AB, Sweden) was performed for further confirmation of *Mycobacterium tuberculosis*.

### 2.3 Statistical analysis

Given the low detection frequency of rare bacteria, statistically significant differences could hardly be identified. Therefore, only the 51 most frequently detected bacterial and fungal species were included for pooled analysis in this study. These 51 microorganisms accounted for 97.35% of all confirmed positive specimens, ensuring the representativeness of the analytical results. Raw data were organized using Microsoft Office Excel 2021 (Microsoft Corporation, USA), and statistical analyses were performed with SPSS 19 (IBM Corporation, USA). Study participants were divided into four age groups in accordance with published criteria [17]: children (0–14 years), young adults (15–47 years), middle-aged adults (48–63 years), and elderly individuals (≥ 64 years). Seasons were classified referring to Chinese standards QX/T 152−2012 and GB/T 42074−2022 based on temperature characteristics: spring (March to May), summer (June to August), autumn (September to November), and winter (December to February of the subsequent year).

The chi-square test was used to compare differences between groups, and the goodness-of-fit test was applied to assess differences within groups. A P value < 0.05 was considered statistically significant.

## 3 Results

### 3.1 Overall distribution of bacteria and fungi

**Fig 1:**
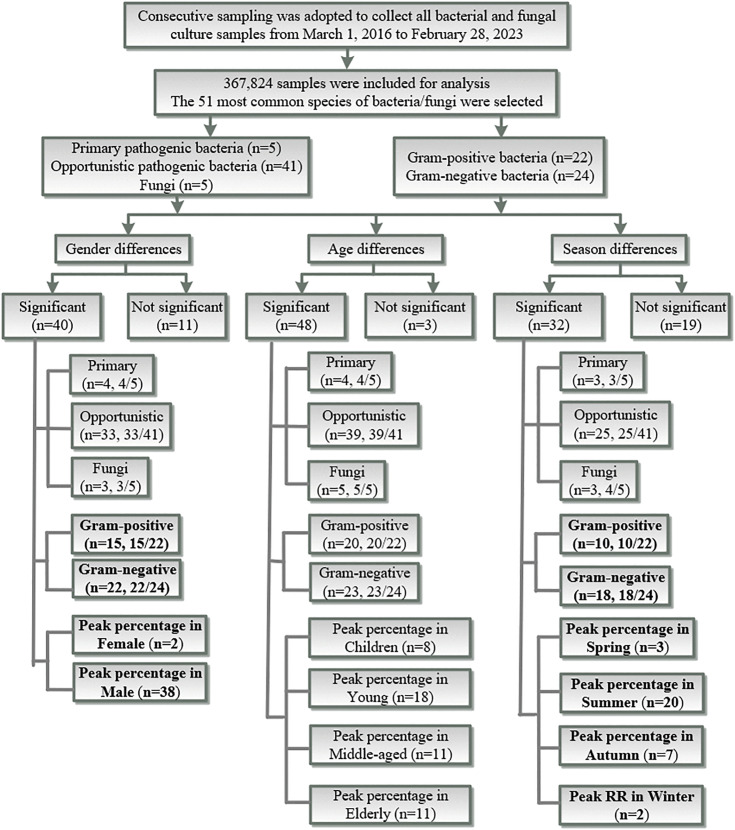
Study flow chart. Note: Bold values indicate significant differences.

The flowchart of the overall analysis is presented in Fig 1.

A total of 367,824 eligible specimens were enrolled in this study. A total of 168 bacterial and fungal species were detected, with 74,838 positive isolates in total. The detailed ranking of detection frequency for each microorganism is shown in Supplementary Material 1. The top 51 bacterial and fungal species included in the subsequent statistical analysis accounted for 97.35% of all positive specimens (72,862/74,838), which demonstrated the representativeness of these 51 microorganisms in clinical infections and laid a solid data foundation for the subsequent analysis of epidemiological characteristics.

### 3.2 Sex-based distribution differences

**Fig 2:**
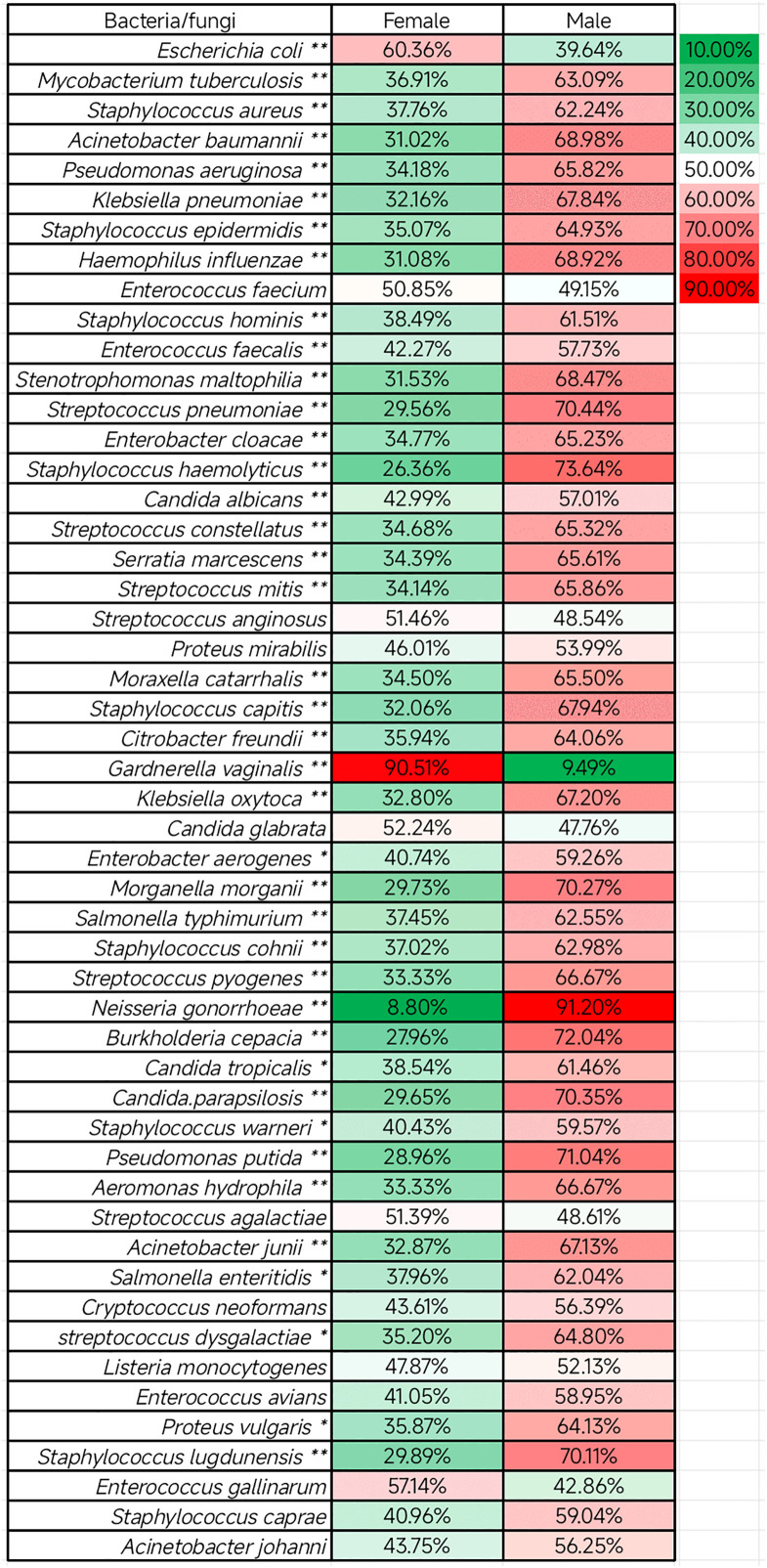
Heatmap of sex Distribution Percentage of 51 Bacterial and Fungal Infections. Note: ** indicates P < 0.001, and * indicates 0.001 ≤ P ≤ 0.05.

Among the 51 predominant bacterial and fungal species, 40 exhibited statistically significant differences in positive detection rates between male and female patients (Fig 2; detailed data are provided in Supplementary Material 2). Stratified by pathogenic types and microbial categories, significant sex differences were observed in 4 out of 5 primary pathogenic bacteria and 33 out of 41 opportunistic pathogenic bacteria. For fungi, 3 out of 5 species showed sex-related disparities. No statistically significant difference was found in the proportion of species with sex differences among the three groups (primary pathogens, opportunistic pathogens and fungi) (χ 2 = 1.11, P = 0.573).

In terms of Gram staining classification, the proportion of bacteria with significant sex-related differences was 68.18% (15/22) for Gram-positive bacteria, which was markedly lower than that of Gram-negative bacteria at 91.67% (22/24) (χ 2 = 4.02, P = 0.045).

Of the 40 microorganisms with distinct sex differences, the number of species with peak detection percentage in male patients (n = 38) was significantly higher than that in female patients (n = 2) (χ 2 = 32.4, P < 0.001).

### 3.3 Age-based distribution differences

**Fig 3:**
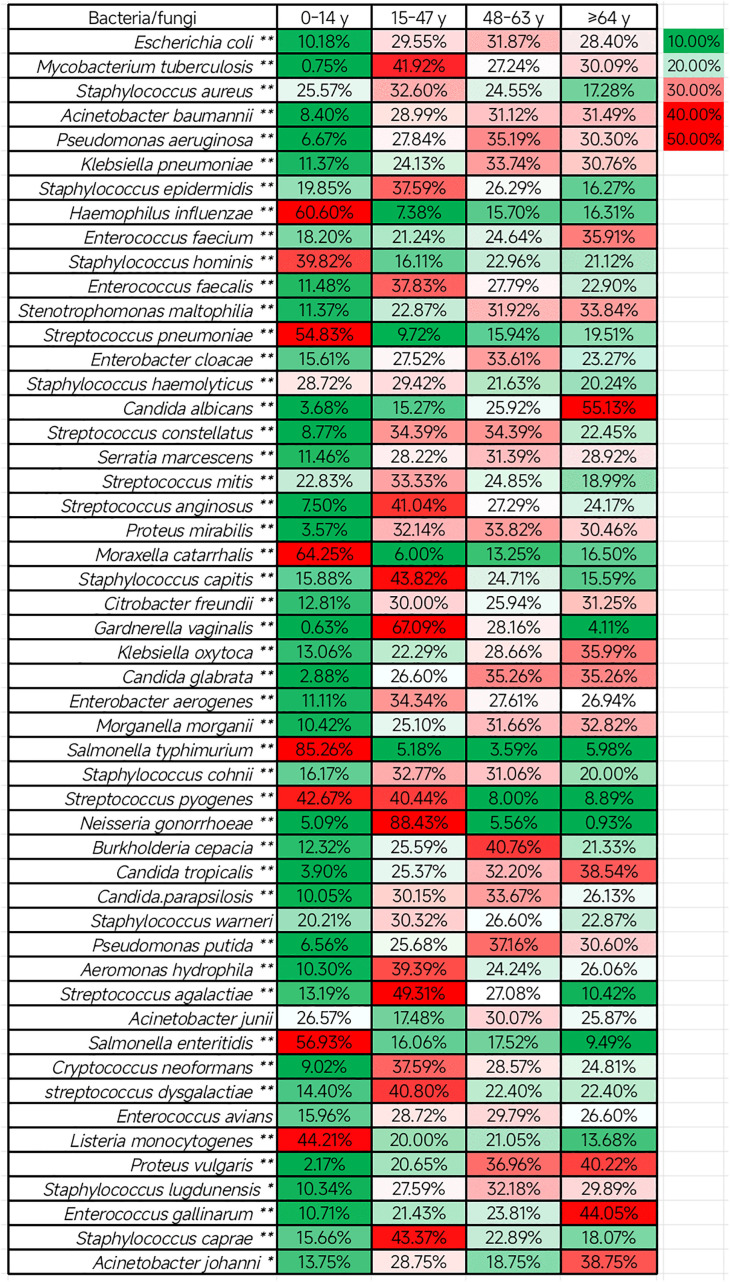
Heatmap of Age Distribution Percentage of 51 Bacterial and Fungal Infections. Note: ** indicates P < 0.001, and * indicates 0.001 ≤ P ≤ 0.05.

Among the 51 predominant bacterial and fungal species, 48 showed statistically significant differences in positive detection rates across different age groups (Fig 3; detailed data are presented in Supplementary Material 3). Stratified by pathogenic types and microbial categories, age-related differences were observed in 4 of the 5 primary pathogenic bacteria and 39 of the 41 opportunistic pathogenic bacteria. All five fungal species exhibited significant age differences. No significant difference was detected in the proportion of species with age disparities among the three microbial groups (χ2 = 2.19, P = 0.335).

According to Gram staining classification, significant age-associated differences in positive rates were found in 20 out of 22 Gram-positive bacteria (90.91%) and 23 out of 24 Gram-negative bacteria (95.83%). There was no statistically significant difference between the two groups (χ2 = 0.46, P = 0.499).

For the microorganisms with notable age-related variations in positive rates, the number of species with peak detection percentage was 8 in the children group, 18 in the young adult group 11 in the middle-aged group, and 11 in the elderly group. No significant difference was observed among these age groups (χ2 = 4.50, P = 0.212).

### 3.4 Seasonal distribution differences

**Fig 4:**
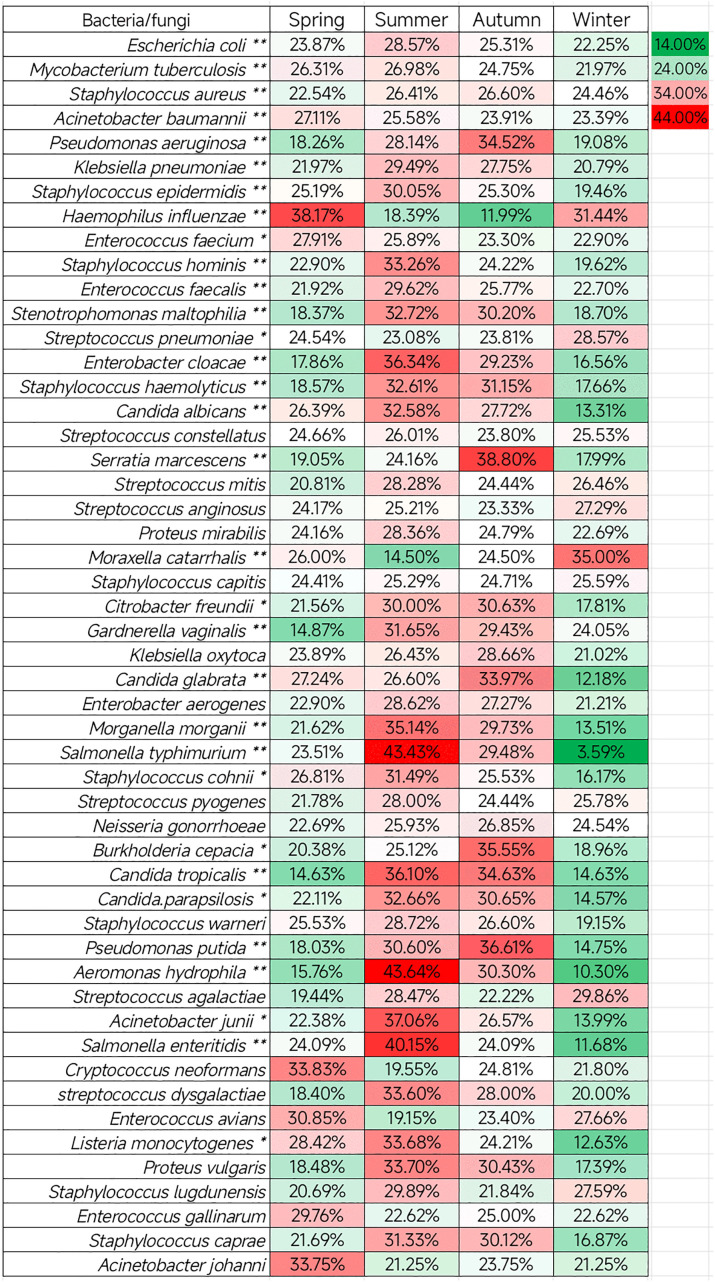
Heatmap of Season Distribution Percentage of 51 Bacterial and Fungal Infections. Note: ** indicates P < 0.001, and * indicates 0.001 ≤ P ≤ 0.05.

Among the 51 predominant bacterial and fungal species, 32 had statistically significant differences in positive detection rates across different seasons (Fig 4; detailed data are shown in Supplementary Material 4). Stratified by pathogenic types and microbial categories, seasonal variations were identified in 3 of the 5 primary pathogenic bacteria and 25 of the 41 opportunistic pathogenic bacteria. Seasonal differences were also observed in 4 out of 5 fungal species. No statistically significant difference was found in the proportion of species with seasonal disparities among the three microbial groups (χ2 = 0.71, P = 0.702).

In terms of Gram staining classification, the proportion of bacteria with significant seasonal differences was 45.45% (10/22) for Gram-positive bacteria, which was significantly lower than 75.00% (18/24) for Gram-negative bacteria (χ2 = 5.26, P = 0.022).

For the microorganisms with remarkable seasonal differences in positive rates, the number of species with peak detection percentage was 3 in spring, 20 in summer, 7 in autumn and 2 in winter. The number of species reaching the peak in summer was the highest, and the difference was statistically significant (χ2 = 25.75, P < 0.001).

## 4 Discussion

The specimens enrolled in this study were collected from patients presenting with suspected infectious symptoms. The distribution of infected patients across sex, age groups and seasons is representative of the epidemiological characteristics of infections. Most previous studies have focused on a single or a small number of predominant bacteria, merely describing the distribution patterns of individual pathogens [10,18,19]. By contrast, the present study integrated distribution data of a wide range of bacteria and fungi, incorporated three key variables including sex, age and season, and further conducted analyses based on pathogenic types and Gram staining classification, thereby establishing a comprehensive understanding of the predilection characteristics of microbial infections. For instance, *Haemophilus influenzae* infections predominantly occur in spring and are more prevalent among male children. This distinct epidemiological pattern can assist clinicians in clinical diagnosis and narrow down the scope of pathogen screening. Furthermore, comparisons of infection distribution characteristics among different pathogenic types (primary pathogens vs. opportunistic pathogens), Gram staining properties (Gram-positive vs. Gram-negative bacteria), and microbial categories (bacteria vs. fungi) provide solid data for exploring the biological traits of bacteria and fungi, and also lay a foundation for further targeted mechanistic research.

Comparisons with the findings of mainstream national antimicrobial resistance surveillance and multicenter studies revealed that the composition of predominant strains identified in the present study was highly consistent with those reported in other regions of China, verifying the good external validity and general applicability of our results. The China Hospital Invasive Bacterial Resistance Surveillance Network has investigated the epidemiological profiles of several major bacterial pathogens. Its data indicated that the five most frequently isolated strains were *Escherichia coli* (19.3%), *Klebsiella* species (14.7%), *Acinetobacter* species (10.1%), Staphylococcus species (9.0%), and *Pseudomonas aeruginosa* (8.7%) [19]. A study conducted in Zhengzhou, China reported that the most prevalent bacteria were *Klebsiella pneumoniae* (17.71%), *Escherichia coli* (14.45%), *Staphylococcus aureus* (7.42%), *Pseudomonas aeruginosa* (6.64%), and *Acinetobacter baumannii* (5.75%) [20]. In our study, the top ten bacterial and fungal species by detection frequency were *Escherichia coli* (16.77%), *Staphylococcus aureus* (12.21%), *Acinetobacter baumannii* (9.32%), *Pseudomonas aeruginosa* (9.23%), *Klebsiella pneumoniae* (7.64%), *Staphylococcus epidermidis* (5.35%), *Haemophilus influenzae* (3.71%), *Enterococcus faecalis* (3.38%), *Staphylococcus hominis* (2.63%), and *Enterococcus faecium* (2.32%). Although slight variations in the proportional distribution were observed due to regional medical settings and differences in study populations, the overall ranking of microbial species was comparable to that in other domestic investigations. These findings further demonstrate that the results of this study are widely referential.

The differences in sex distribution indicated that bacterial and fungal infections exhibited an obvious predilection for male patients. Moreover, sex exerted a significantly stronger impact on Gram-negative bacteria than on Gram-positive bacteria, which provides new evidence for studies on sex-related immune discrepancies and pathogen recognition mechanisms. Previous studies have reported that the incidence of *Escherichia coli* bacteremia is higher in females [21], while males are more susceptible to infections caused by *Acinetobacter baumannii* and *Neisseria gonorrhoeae* [22–24], and these findings were validated in the present study. There exists a general consensus that females possess more robust immune function than males, though a small number of studies hold opposing views [25,26]. In this study, the percentage peaks of bacterial and fungal infections were far more frequently observed in males than in females, further supporting the viewpoint of superior immune function in females. Gram staining categorizes bacteria based on cell wall structures. Gram-positive bacteria have a thick, single-layered cell wall that is loosely attached to the cell membrane, whereas Gram-negative bacteria feature a multi-layered cell wall consisting of an inner layer tightly bound to the cell membrane and an outer membrane [27]. In accordance with this classification, our results revealed that a larger proportion of Gram-negative bacteria infection presented significant sex-associated disparities compared with Gram-positive bacteria. This suggests that Gram staining characteristics may serve as a key factor contributing to sex differences in bacterial infections, which is presumably attributed to variations in host immune recognition efficiency and tissue colonization capacity resulting from distinct cell wall architectures between the two groups of bacteria. In contrast, pathogenicity (primary pathogens versus opportunistic pathogens) was not a major determinant of such sex disparities.

Stratified analysis by age revealed that most bacterial and fungal infections displayed age predilection, while Gram staining classification and pathogenic characteristics were not the primary determinants of such age-related differences. The age-specific infection patterns were more closely associated with behavioral and physiological traits [21–23,28, 29]. Our results demonstrated no statistically significant difference in the proportion of species with age disparities between Gram-positive and Gram-negative bacteria. Likewise, no significant differences were observed among opportunistic microorganisms, primary pathogens and fungi. These findings indicate that Gram staining profiles and pathogenic properties exert limited effects on age-related variations in infections. It is generally acknowledged that children and the elderly have weaker immune function compared with young and middle-aged adults [30,31]. Nevertheless, in the present study, the peaks of age-specific positive percentage were most frequently detected in the young adult group, rather than in children or the elderly. Although this trend did not reach statistical significance, the underlying reasons warrant further exploration. Specifically, the high detection rates of *Gardnerella vaginalis* and *Neisseria gonorrhoeae*, which presented the highest percentage in the young adult group, may be linked to sexual activity [22].

Distinct seasonal distribution patterns were observed, with bacterial and fungal infections peaking predominantly in summer. This trend differs markedly from viral infections, which are more prevalent in winter and spring [32]. In addition, Gram-negative bacteria were more susceptible to seasonal temperature fluctuations, a finding with important implications for clinical differential diagnosis [33]. Previous studies have demonstrated that viral infections occur more frequently during winter and spring [34,35]. In the present study, the number of microbial species with distinct percentage variations in positive rates was the highest in summer, indicating a clear divergence in the peak activity seasons between bacterial/fungal pathogens and viruses. This characteristic can facilitate the preliminary differentiation of infection types in clinical practice and guide timely adjustment of empirical antimicrobial regimens. In terms of environmental resistance, it has been reported that fungi exhibit stronger tolerance to dry conditions than bacteria [36]. However, our results showed that the extent to which fungal infections were affected by ambient temperature was comparable to that of bacterial infections. Gram-negative bacteria were more sensitive to seasonal temperature changes than Gram-positive bacteria, suggesting that the thick cell wall of Gram-positive bacteria may confer greater resistance to temperature fluctuations, thereby supporting their survival and proliferation. Combined with our previous finding that Gram-negative bacterial infections are more prone to sex-related disparities, this study provides new insights for future research on the molecular mechanisms underlying environmental adaptability of Gram-positive and Gram-negative bacteria.

Collectively, the sex-, age-, and season-specific pathogen distribution identified here supports stratified infection prevention strategies, as well as hospital antimicrobial stewardship and seasonal surveillance. Sex- and age-tailored screening and intervention can be implemented for high-risk populations, while enhanced environmental control is recommended in summer when most Gram-negative pathogens peak. For antimicrobial stewardship, clinicians may select stratified empirical antibiotics based on patient demographics and seasonal pathogen trends to reduce overuse of broad-spectrum agents. Meanwhile, intensified summer monitoring focusing on temperature-sensitive bacteria and fungi can be integrated into routine seasonal surveillance to detect infection clusters early [37].

This study has several limitations. First, due to the limited data of rare pathogens which hard to yield statistically significant results, only the 51 most frequently detected bacterial and fungal species were included in the analysis, while rare microorganisms were not enrolled. Second, all specimens were collected from symptomatic outpatients and inpatients, with no stratification by clinical manifestations or admission status. While our pooled data reflect the overall infection landscape of a tertiary hospital, inpatients exhibit more complex infection and resistance profiles associated with nosocomial exposure, invasive interventions and intensive antibiotic pressure. Accordingly, the observed demographic and seasonal pathogen patterns integrate two distinct patient subgroups, which may introduce bias when extrapolated to outpatients or inpatients alone. Third, data regarding bacterial antimicrobial resistance of the enrolled patients were not analyzed in this study. Fourth, this is a single-center retrospective study, which may limit generalizability to other geographic regions.

## 5 Conclusions

This study summarized the sex, age and seasonal predilection characteristics of infections caused by the 51 most prevalent bacterial and fungal species. Notably, Gram-negative bacteria were more susceptible to sex factors and seasonal temperature variations compared with Gram-positive bacteria. In addition, females exhibited stronger resistance to bacterial infections than males, suggesting the presence of sex-related differences in immune responses. Bacteria and fungi were most active in summer, highlighting the impact of environmental factors on the prevalence of pathogens. These findings indicate that the above distribution characteristics should be taken into account when formulating strategies for infection prevention, control, diagnosis and treatment.

## Supporting information

Supplementary Material 1 Supplementary Material 1 ranking of all 168 bacteria or fungi. (XLSX)

Supplementary Material 2 Detailed sex distribution characteristics of bacterial and fungal infections. (XLSX)

Supplementary Material 3 Detailed age distribution characteristics of bacterial and fungal infections. (XLSX)

Supplementary Material 4 Detailed Seasonal distribution characteristics of bacterial and fungal infections. (XLSX)
